# How early life respiratory viral infections impact airway epithelial development and may lead to asthma

**DOI:** 10.3389/fped.2024.1441293

**Published:** 2024-08-02

**Authors:** Sergejs Berdnikovs, Dawn C. Newcomb, Tina V. Hartert

**Affiliations:** ^1^ Department of Medicine, Northwestern University Feinberg School of Medicine, Chicago, IL, United States; ^2^ Department of Medicine, Vanderbilt University Medical Center, Nashville, TN, United States; ^3^ Department of Pediatrics, Vanderbilt University Medical Center, Nashville, TN, United States

**Keywords:** asthma, airway epithelium, development, respiratory virus, metabolism

## Abstract

Childhood asthma is a common chronic disease of the airways that results from host and environment interactions. Most risk factor studies of asthma point to the first year of life as a susceptibility window of mucosal exposure that directly impacts the airway epithelium and airway epithelial cell development. The development of the airway epithelium, which forms a competent barrier resulting from coordinated interactions of different specialized cell subsets, occurs during a critical time frame in normal postnatal development in the first year of life. Understanding the normal and aberrant developmental trajectory of airway epithelial cells is important in identifying pathways that may contribute to barrier dysfunction and asthma pathogenesis. Respiratory viruses make first contact with and infect the airway mucosa. Human rhinovirus (HRV) and respiratory syncytial virus (RSV) are mucosal pathogens that are consistently identified as asthma risk factors. Respiratory viruses represent a unique early life exposure, different from passive irritant exposures which injure the developing airway epithelium. To replicate, respiratory viruses take over the host cell transcriptional and translational processes and exploit host cell energy metabolism. This takeover impacts the development and differentiation processes of airway epithelial cells. Therefore, delineating the mechanisms through which early life respiratory viral infections alter airway epithelial cell development will allow us to understand the maturation and heterogeneity of asthma and develop tools tailored to prevent disease in specific children. This review will summarize what is understood about the impact of early life respiratory viruses on the developing airway epithelium and define critical gaps in our knowledge.

## Introduction

Childhood asthma is a common chronic disease of the airways that results from host and environment interactions (1–5). Most risk factor studies of asthma point to the first year of life as a critical susceptibility window of mucosal exposure that directly impacts the airway epithelium and has the capacity to reshape its development (6–8). Respiratory viruses are a consistently identified asthma risk factor and are also associated with childhood asthma exacerbations (9, 10). Identifying the impact of early life environmental asthma risk factors, such as respiratory viral infection, on the developing airway epithelial cells (AECs) and defining critical windows of susceptibility to infection will ultimately allow us to better understand the development and heterogeneity of asthma and to create tools tailored to prevent disease in specific children, or to treat specific asthma phenotypes. We recognize that other factors including host and viral genetic factors including gene-environment interactions contribute to the variable susceptibility and range of developmental responses to infection that may contribute to wheeze and asthma (11–13). However, this review focuses on the impact of early life respiratory viruses, particularly respiratory syncytial virus (RSV), on airway epithelial development and defining remaining gaps in our knowledge.

## Postnatal development of human airway epithelium in health and asthma

**Figure 1:**
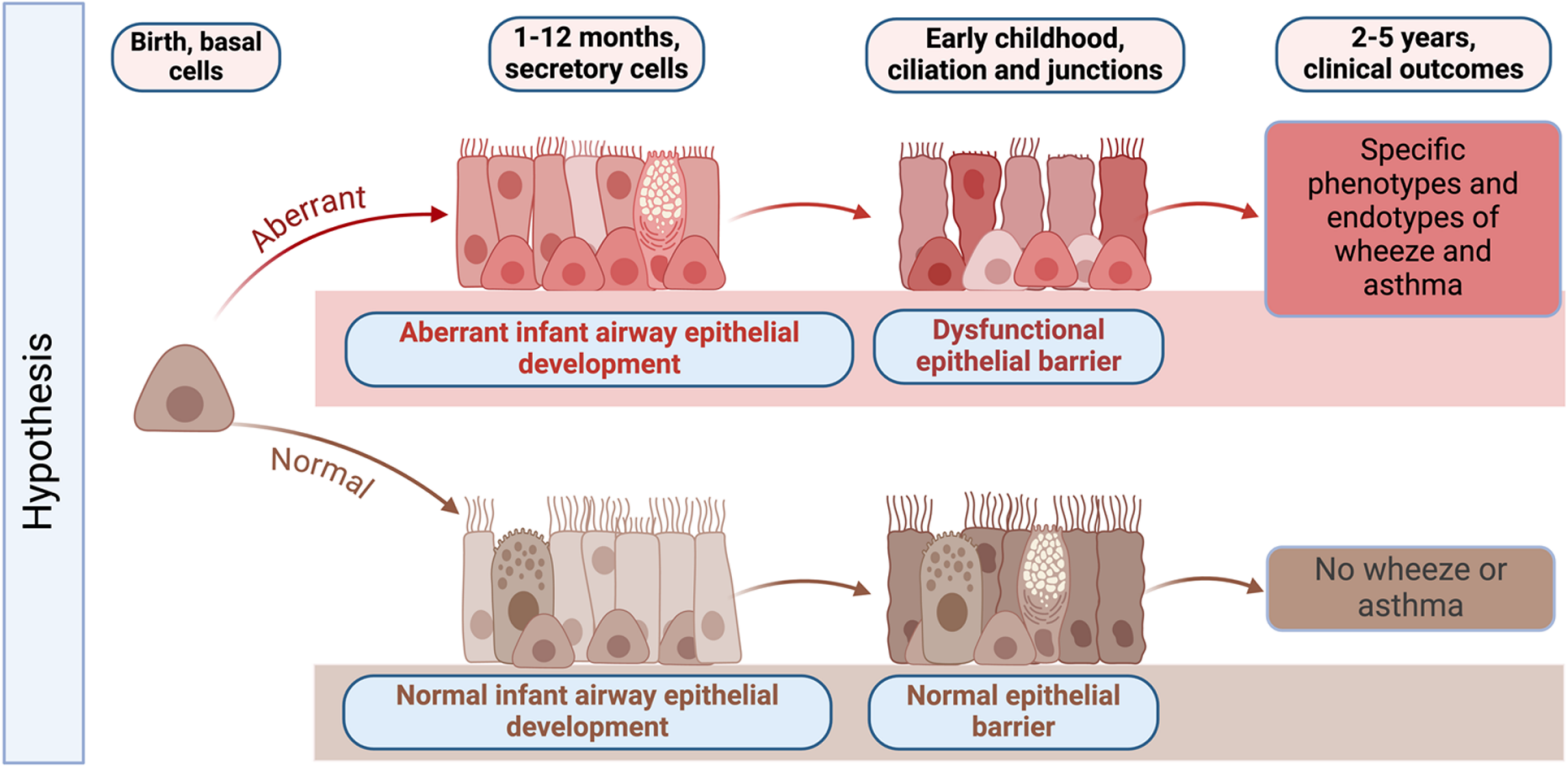
Graphical abstract of hypothesized changes in the development of the early life airway epithelium in health and disease. Infancy is a critical time window during which host genetics, environmental exposures such as respiratory viruses, and epigenetic modifications may result in developmental and metabolic reprogramming of airway epithelium. Developmental reprogramming during infancy leads to the formation of dysfunctional epithelial barrier, which may promote the development of specific phenotypes of wheeze and asthma later in life. Created with BioRender.com.

Unlike other organs, the airway and lung are not fully developed at birth and continue to develop postnatally. The AEC barrier and its function in childhood is shaped and regulated by active on-going developmental programs, with morphogenesis of AECs continuing after birth (14–16). AEC differentiation occurs largely in the first year of life but continues until approximately 2 years of age. After birth, AEC morphogenesis continues in both the upper and lower airway concurrent with additional branching and expansion of the alveoli (14–16). From birth to 2 months of age we have shown that AECs have a basal cell predominant phenotype with fewer mucociliary and ciliated cells compared with mature airway epithelium (17). AEC differentiation and mucociliary lineage specialization increase dramatically and linearly in the first two years of life, which culminates in full lung maturation by 3 years of age (18) (Figure 1). During this process, basal and suprabasal cells give rise to club and tuft cells. Tuft cells can further differentiate either into ionocytes or neuroendocrine cells, while club cells give rise to ciliated epithelial cells via deuterosomal intermediates, or mucus-producing goblet cells (19). Consequently, perturbation of barrier morphogenesis in early life may have a lasting effect on adult epithelium via developmental reprogramming (20–22). Given this developmental sequence, Figure 1 outlines our hypothesis that infancy is a critical time window during which developmental and metabolic reprogramming of airway epithelium may be altered sequentially during development. This developmental reprogramming is likely driven in part by host genetics and epigenetic modifications, but also by early life mucosal exposures such as respiratory viral infections that may lead to epigenetic changes and other pathways that may drive aberrant airway epithelial development. In the subsequent sections, we discuss the different pathways that are aberrant in AECs from children with early-life RSV infections and how this altered development results in decreased airway barrier function and increased RSV infectivity in *in vitro* cultured NAECs (17).

Studying the human airway epithelium is challenging. Only human longitudinal studies can establish the sequence of airway epithelial development, identify changes over time, and provide insight into cause-and-effect relationships. Prospective longitudinal studies are expensive, challenging, time consuming, and most are limited to the use of upper airway epithelium to understand development. In children, nasal AECs are used because collection of bronchial airway cells is highly invasive; however, studies reveal similar developmental trajectories that allow use of nasal AECs to study airway epithelial development (23–25). The nasal transcriptome has been demonstrated to be an excellent and well-accepted proxy of expression changes in the lung airway transcriptome in asthma, as well as in distinguishing phenotypes of asthma (23–25). This provides support for use of nasal airway epithelium to understand the developmental origins of asthma, to determine the trajectory of abnormal and normal AEC development in asthma and in health, and how early life environmental exposures, such as respiratory viral infections, alter NAEC metabolism, development, and barrier function.

Little is known about how early life AEC development affects risk of childhood asthma and if AEC developmental trajectories are sustained through life. Most studies of AECs are cross-sectional studies that sample children greater than 3 years of age or adults. These studies show that nasal and bronchial AECs from subjects with asthma have altered differentiation patterns and deficient barrier function with increased susceptibility to injury and deficient repair (26–28). A cellular single cell RNA-seq (scRNA-seq) census of human lower airways from adult patients with asthma (ages 45–60) showed that mucous and goblet cell hyperplasia stems from a novel mucous ciliated cell state (29). Another study of AECs from adults with asthma (ages 18–50) revealed transcriptional programs and cell subsets specific to allergic asthma, including hillock cells, a cell type first described in 2019 which is a transitional cell between basal and club cells (30, 31). Additionally, AECs from adults with asthma that had persistent wheeze at 3 years of age had increased gene expression in extracellular matrix and adhesion pathways compared to AECs from adults with asthma that did not have persistent wheeze at 3 years of age (32). Our report of data mining of publicly available adult asthma AEC datasets determined that developmental pathways of WNT, Notch and ephrin signaling were dysregulated (28). Other studies have demonstrated dysregulated WNT signaling in AECs and fibroblasts from patients with asthma (33, 34), and loss of ephrin signaling in AECs has also been linked to increased Type 2 cytokine expression in patients with asthma (35). Additionally, childhood-onset asthma has been reported to be characterized by airway epithelial hillock-to-squamous differentiation in early life (17, 36) and Notch/Jagged pathway dysregulation was found to contribute to deficient repair in AECs of children with wheeze (37). Our recent study using scRNA-seq to characterize differentiation status of nasal AECs from 2 to 3 year old children found that epithelium from children with wheeze is characterized by an early activity of WNT and Notch/Jagged developmental pathways in basal cells and delayed onset of maturation of early epithelial progenitors and club cells (17). Such aberrant developmental processes and reshaping of epithelial subsets seem to be congruent between adult asthma and wheeze epithelial phenotypes reported in children (38), suggesting an early or potentially common epithelial setpoint in the development of asthma.

## Respiratory viruses and childhood asthma

The association of respiratory viral infection and the development of asthma has been recognized for decades, particularly the association with respiratory syncytial virus (RSV) and human rhinovirus (HRV) infections (39). RSV is the most common cause of infant acute respiratory infection as well as the single major cause of hospitalization and respiratory mortality during infancy (40, 41). While nearly all children are infected with RSV by the age of 2, approximately half become infected during the first year of life as determined by serology (7, 42–45). RSV is a seasonal mucosal pathogen that infects the ciliated respiratory epithelium of the upper and lower airway as a descending infection, causing disease of variable severity (46, 47). At early ages, most primary RSV infections cause infection of the lower airway epithelium, however, a very small minority have severe disease or result in hospitalization, an estimated 1–3% of infected infants (46, 48–50). The first infection is generally the most severe infection, and illness severity becomes less common with advancing age (43, 51, 52). In addition to being a common respiratory pathogen, RSV is also the most common cause of serious infant respiratory morbidity and mortality and a consistently identified asthma risk factor with a high population attributable fraction (6). In support of a critical susceptibility period during early life in which RSV infection has a greater impact on asthma development, we have previously demonstrated an age-dependent association between RSV infection and asthma risk. Children with delayed RSV infection until after the first year of life, compared with infants infected during the first year of life, have a significantly decreased risk of wheeze and asthma through age 5 years (7). Additionally pointing to a critical susceptibility period, asthma incidence decreases over time following infant RSV infection, however, the effect of at least early life RSV LRTI persists through adolescence and early adulthood demonstrated in longitudinal cohort studies by diagnosis, lung function and image-related changes into early adulthood (53–56). Additionally, in *in vitro* RSV infection of differentiated nasal AECs (NAECs) in culture, NAECs from children with RSV infection prior to age 1 and wheeze at age 3 had decreased NAEC barrier function and increased RSV viral gene expression compared to children that had RSV after age 1 and no wheeze at age 3 (control samples) (17). These data support that RSV infection during the first year of life is associated with altered AEC development and increased risk of wheeze and asthma. While the exact timing of infection during the first year of life and its impact on the development of asthma is unknown, we hypothesize that this is a critical period of airway epithelial development and infection during this time results in inappropriate or dysregulated responses to respiratory viruses that are pathologic and contribute to short-and long-term effects on AEC development and barrier function (57). Details of these studies are discussed in the subsequent section.

Human rhinovirus (HRV) infects all infants at least once in the first year of life, and wheezing or lower respiratory tract illness, in contrast to RSV, increases with advancing child age (51, 58). HRV lower respiratory tract infections (LRTI) are associated with the development of asthma and are associated with a large proportion of asthma exacerbations in children and adults (59–62). Studies of *in vitro* infection of AECs from patients with asthma demonstrate mucus hypersecretion, goblet cell hyperplasia, and remodeling phenotypes including induction of epithelial-to-mesenchymal transition, and fibroblast-to-myofibroblast transdifferentiation, a response which is more exaggerated in asthmatic AECs (63–65). Additionally, a comparison of AECs from adults with asthma that had persistent wheeze or not in the first 6 years of life showed that AEC cultures infected with a group A HRV had increased expression of genes in the toll-like receptor pathway, the IFN pathways, and *IL12* and *IL8* genes compared to AECs from adults that did not have persistent wheeze in early life (32). However, how HRV infections in infancy impact AEC development, differentiation, and barrier function, remains unclear. Asymptomatic HRV infections are common and are difficult to detect by serology, making it challenging to identify the timing and frequency in HRV infections in infants and young children. However, extrapolating from animal models and *in vitro* studies, HRV results in changes in airway epithelial development and barrier, and repeated infections, common in early life, may result in persistence of these changes.

The mechanisms by which an acute, early-life respiratory viral infection causes long-term pulmonary effects in humans and the subsequent development of asthma, however, remain poorly understood (53, 57, 66–69). Since AEC development continues from birth until age 3, early-life mucosal exposures may alter the course of AEC differentiation and contribute to the development of asthma. Respiratory viruses can injure as well as alter the host cell transcriptional and translational processes, a process known as “molecular hijacking”, and induce epigenetic and metabolic changes. Respiratory viruses are additionally associated with distinct changes in the airway microbiome commensal bacterial communities that have been repeatedly associated with the development of asthma (70, 71). Consequently, respiratory viruses are an early life mucosal exposure that have the potential to alter AEC development and barrier function, which may translate to increased susceptibility to aeroallergens or pollutants and an increased risk of developing asthma (72, 73). However, it is important to acknowledge that the development of asthma is multi-factorial and early-life respiratory viral infections are one of many risk factors for the development of asthma. Other risk factors, including maternal exposures, host and viral genetics, gene by viral interactions, and exposure to other environmental pollutants/factors are also important contributors to the development of asthma. Additionally, RSV infection may differentially contribute to specific phenotypes and endotypes of asthma and assessing asthma as an umbrella diagnosis may attenuate the effects that might be identified if studying the contribution of infection to specific asthma phenotypes. In secondary analyses we have demonstrated that RSV contributes to a predominantly non-allergic asthma phenotype in children (7). Longitudinal, prospective studies that assess the effect of respiratory viral infections and include serial AEC sampling starting at birth are required to answer these questions.

## Viral reprogramming of airway development via impact on epithelial differentiation and epigenetic modification

RSV infections trigger transcriptomic and proteomic changes in host cells, which include initiation of the anti-viral IFN response, endoplasmic reticulum stress, oxidative stress, and programmed cell death. These RSV-induced host cellular responses in AECs can lead to airway remodeling and initiation of airway inflammation (74). Upon infection, viral proteins transform AECs by inducing alterations in the normal cell growth and differentiation pathways. While human papillomaviruses and other viruses cause extreme transformation of epithelial cells and promote carcinomas (75, 76), the effect of respiratory viruses on AEC differentiation pathways is not as substantial. For example, RSV infects basal AECs in culture and alters AEC differentiation resulting in a decrease of formation of ciliated cells and an increase in secretory epithelial cells during differentiation (77). Another report showed that RSV infection of bronchial epithelial cells *in vitro* dysregulated Notch/Jagged signaling and co-culture of CD4+ T cells with infected cells promoted Type 2 cytokine production (78). *in vivo*, RSV infection in neonatal mice exacerbated an allergic asthma phenotype by increasing lung eosinophils and Type 2 cells (79).

It has been increasingly recognized that barrier disruption in asthma is durable and may persist beyond a simple inflammatory insult (80–83). AEC dysfunction persists after removal of cells from an *in vivo* inflammatory environment indicating a lasting reprogramming effect that can be studied *in vitro*. Such reprogramming of epithelium strongly suggests epigenetic modification (28, 84–88). Epigenetic mechanisms are thought to play a fundamental role in the long-term sequelae after RSV infection, perhaps enhanced by the persistence of or response to infection and resulting in different phenotypes observed (89, 90). Therefore, respiratory viral infections in early life may alter airway epithelial development and differentiation, providing a potential mechanism for increased wheeze and asthma.

## Respiratory viral infection and reprogramming of epithelial metabolism

Epithelial development is also sensitive to metabolic changes, especially early in life. Changes in metabolism have consequences for both establishment of competent epithelial barrier and immune response (91–94). Despite the growing recognition of immunometabolism in homeostasis and disease (95), there are very few studies examining the contribution of AEC metabolism to allergic disease pathogenesis (96–98), and almost none in humans. Perturbation of AEC metabolism in infancy may have lasting effects on AEC differentiation via alteration of fundamental developmental or differentiation programs and/or epigenetic reprogramming, since metabolism is a powerful modulator of epigenetic regulatory mechanisms (99). Such perturbations have the potential for lasting epithelial barrier dysfunction that may render the airway more susceptible to allergic sensitization.

Viruses themselves are metabolically inert and must rely on metabolic events in the cell to generate their component parts and to replicate new viral copies. Frequently, the cell at the time of infection is in a quiescent state, but the infection acts to change the cell's metabolism (100, 101). Many metabolic pathways in a host cell such as glycolysis, amino acid and nucleotide synthesis are altered following virus infection. During infection, viral proteins interact with various cellular glycolytic enzymes, and this interaction enhances the catalytic framework of the enzymes and subsequently the glycolytic rate of the cell (102). During RSV infection, amino sugars, nucleotide sugars and palmitic acid were found to be more abundant compared with the levels observed in non-infected cells, which allows post-translational protein modification necessary for the maturation of several RSV proteins (103). Additionally, increased levels of oxidized glutathione and polyamines were associated with oxidative stress in RSV infected cells (103, 104). Such host cell metabolic changes ensure the energy and building blocks necessary for virus replication (103, 105). We have previously reported that RSV infection in infancy is associated with metabolic reprogramming of nasal AECs later in life (2–3 years of age) (67). This metabolic reprogramming was characterized by significant increase in glucose uptake, differential utilization of glucose by AECs and altered preferences for metabolism of several carbon and energy sources, with RSV-induced metabolic changes most pronounced in male airway epithelium (67). Strikingly, these metabolic alterations were measured in absence of active RSV infection, implying epigenetic or “metabolic memory” that may be persisting in epithelial cells developmentally reprogrammed in the first year of life.

**Figure 2:**
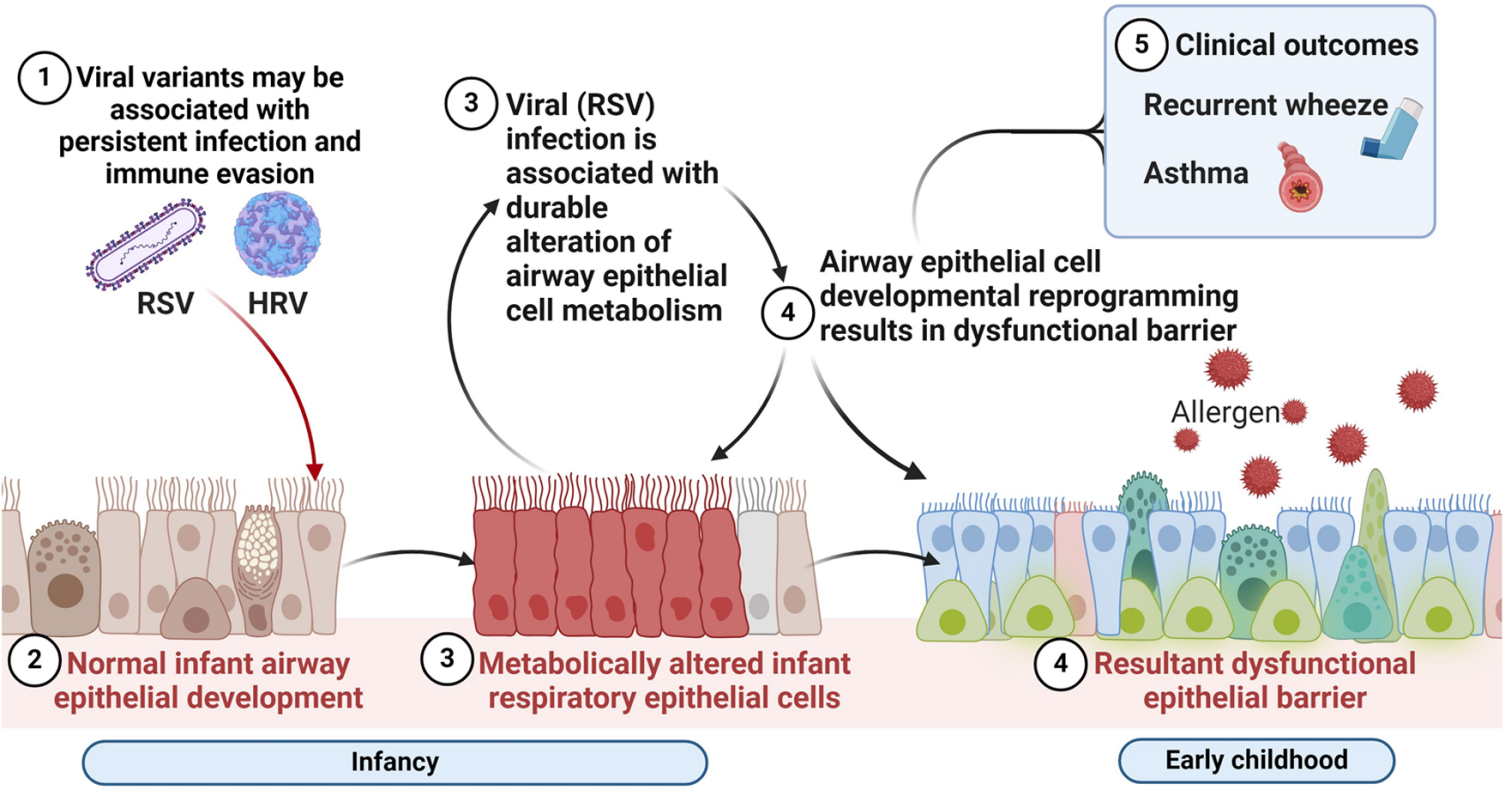
Respiratory virus mediated alterations in the airway mucosal niche in early life associated with chronic respiratory outcomes of wheeze and asthma. We hypothesize that alterations in airway epithelial cellular metabolism and developmental reprogramming associated with early mucosal respiratory viral infection may lead to barrier dysfunction, enhanced susceptibility to recurrent infections and other asthma risk factors and increased risk of wheeze and asthma. Created with BioRender.com.

Consequently, perturbation of AEC metabolism in infancy may have lasting effects on AEC differentiation via developmental or epigenetic reprogramming. As shown in Figure 2, these are the hypothesized alterations in AEC metabolism resulting from early mucosal respiratory viral infection that may lead to barrier dysfunction, enhanced susceptibility to other asthma risk factors and increased risk of wheeze and asthma. However, the metabolic pathways promoting this aberrant developmental program in the early life origins of asthma are not understood, nor are the specific effects of early life respiratory viruses on airway metabolism.

## Metabolism and anti-viral responses: the chicken or the egg debate of the causal role of respiratory viral infection in the development of asthma

In the sections above we described examples of how respiratory viruses can affect the developmental trajectory of AECs via transcriptional, epigenetic and metabolic mechanisms, thus promoting dysfunctional epithelial barrier and predisposing to development of asthma. However, the converse is also true. Epithelial cells undergoing transcriptional, epigenetic and metabolic change in early life development for reasons other than viral infection (other environmental insults, hormonal imbalances, nutrition, systemic dysbiosis, genetics) may render the host more susceptible to recurrent or more severe respiratory viral infections. Ample evidence shows that such processes may result in deficient host antiviral responses, metabolic conditions favoring successful infection, or relative increase in epithelial cell subsets prone to initial infection with virus.

We have shown that downregulation of the insulin receptor (INSR) signaling pathway and loss of differentiation in AECs are conserved features of asthma in adults and children (28). This was evidenced by the downregulation of the insulin target genes *INSR* and *IRS2*, decrease in expression of pyruvate metabolism markers, as well as changes in mitochondrial respiratory chain genes (28, 106). There is also a demonstrated bioenergetic switch from glycolysis to arginine metabolism in the mitochondria of asthmatic AECs (107, 108). Further, there is growing evidence for dysregulated carbohydrate metabolism in inflammatory conditions, including asthma. High fructose containing foods are associated with asthma, possibly because of the high fructose:glucose ratios which may relate to glucose utilization by the early life developing AEC (109, 110). Similarly, high sucrose diets are associated with increased eosinophil cytokine content and airway resistance in allergen-challenged mice (111). We have also shown that energetic glucose consumption is altered in nasal AECs from children with wheeze (112). Moreover, using single cell RNA-sequencing (scRNA-seq) profiling of nasal AECs from children with wheeze and infant RSV infection, we found that wheeze epithelium alone (in absence of infant RSV) has a distinct developmental phenotype characterized by overactivation of basal cells, expansion of club precursors, decreased expression of anti-viral genes, increase in expression of receptors for RSV and HRV and increased susceptibility to RSV infection *in vitro* (17).

Manipulating metabolism *in vivo* has been demonstrated to reduce the infectivity of respiratory viruses, including SARS-CoV2 (101). Manipulating glucose metabolism during different stages of viral infection can have either detrimental or beneficial effects (113). Glucose and lipid metabolism are known to directly regulate type I IFN production (114), and targeting metabolic pathways is useful in promoting antiviral immunity via modulation of type I IFNs or cholesterol metabolism (115). High glucose is known to suppress IFN expression, which is linked to compromised host defense against infection in diabetes (116). In mouse models, fasted mice supplemented with low glucose showed higher IFN-β production after vesiculovirus infection compared to animals supplemented with high glucose. Low glucose supplemented animals also had lower viral replication, suggesting that downregulated glucose metabolism promoted the type I IFN response and antiviral response (117). Conversely, IFNα therapy for chronic hepatitis C has been shown to impair glucose tolerance in non-diabetic patients (118), supporting the fundamental reciprocal relationship between energy metabolism and anti-viral pathways.

Loss of differentiation resulting from metabolic or epigenetic alterations may also render airway epithelium more sensitive to viral insults. For example, experiments in bovine airway epithelial cells showed that differentiated undisturbed mature ciliated cells were more resistant to bovine RSV infection than injured less differentiated epithelial cells found deeper in the epithelial layer and exposed during injury (119). Our recent scRNA-seq findings also show increased expression of RSV receptors in reprogrammed club and secretory epithelial cell subsets from children with wheeze rather than in ciliated mature cells (17). Collectively, the reciprocal cross-regulatory relationship between viral, metabolic and developmental pathways opens up many questions about causality of early life events leading to asthma, with high likelihood of combination of developmental susceptibility and exposures co-occurring during sensitive time frames in postnatal formation of mature epithelium.

## Summary

Understanding development of the airway epithelial barrier after birth through early childhood is key to unraveling the developmental origins of childhood asthma. However, there remain significant challenges in collecting clinically inaccessible tissues and studying the human airway epithelium longitudinally. Several longitudinal birth cohorts have established longitudinal sampling of the airway that are likely to advance our understanding of airway epithelial development and the role of the environment in the perturbation of normal development (120–124). Changes in host metabolism and metabolic reprogramming of epithelium are integral parts of asthma pathogenesis. Studies of epithelial metabolism in the postnatal developmental time frame will likely provide the necessary mechanistic systems biology framework for understanding upstream triggers of developmental reprogramming on gene regulatory/epigenetic level. Longitudinal integration of single cell transcriptomics, metabolism, epigenetic and environmental exposure data as a systems biology approach using primary airway epithelium will likely further advance our understanding of normal and aberrant airway epithelial development. As there are currently no effective primary preventive interventions for asthma, identifying the timing and pathways driving airway epithelial development may inform novel targets for prevention and treatment approaches that regulate the normal and disease-related development of the early life airway epithelium.
